# Coincident-site lattice matching during van der Waals epitaxy

**DOI:** 10.1038/srep18079

**Published:** 2015-12-14

**Authors:** Jos E. Boschker, Lauren A. Galves, Timur Flissikowski, Joao Marcelo J. Lopes, Henning Riechert, Raffaella Calarco

**Affiliations:** 1 Paul-Drude-Institut für Festkörperelektronik, Hausvogteiplatz 5-7, 10117 Berlin, Germany

## Abstract

Van der Waals (vdW) epitaxy is an attractive method for the fabrication of vdW heterostructures. Here Sb_2_Te_3_ films grown on three different kind of graphene substrates (monolayer epitaxial graphene, quasi freestanding bilayer graphene and the SiC (6√3 × 6√3)R30° buffer layer) are used to study the vdW epitaxy between two 2-dimensionally (2D) bonded materials. It is shown that the Sb_2_Te_3_ /graphene interface is stable and that coincidence lattices are formed between the epilayers and substrate that depend on the size of the surface unit cell. This demonstrates that there is a significant, although relatively weak, interfacial interaction between the two materials. Lattice matching is thus relevant for vdW epitaxy with two 2D bonded materials and a fundamental design parameter for vdW heterostructures.

The ability to create heterostructures of 2-dimensionally (2D) bonded materials^1^ has opened up a new direction in condensed matter physics. The large interest in these so called van der Waals (vdW) heterostructures is due to the expectation to observe unusual and intriguing physical properties when 2D bonded materials with special properties, such as mass less Dirac fermions^2^, are stacked on top of each other^3^. Recent examples include the observation of a tunable metal-insulator transition^1^ and the study of strong Coulomb drag^4^ in graphene-boron-nitride heterostructures. Furthermore, the demonstration of improved switching characteristics of phase change memory based on chalcogenide superlattices^5^ and fabrication of light emitting diodes consisting of vdW heterostructures^6^, hint at the possibilities for exploiting such heterostructures in (opto)electronic devices.

VdW epitaxy^7^ is an attractive method for the fabrication of such heterostructures, because one can benefit from the increased purity offered by ultra high vacuum systems, it is scalable and compatible with CMOS technology. In addition this approach makes it possible to fabricate vdW heterostructures that are unstable in air and materials grown on top of each other can also have an intrinsic alignment, which is challenging to achieve by combining individual sheets of 2D materials. However, the growth of 2D materials is distinct from the growth of 3-dimensionally (3D) bonded materials and it is thus not possible to directly apply the epitiaxial rules developed for 3D materials to the growth of 2D materials. For example, it was shown that Sb_2_Te_3_, a typical 2D bonded material, can form coincidence lattices with reconstructed Si(111) surfaces^8^. This was attributed to the small interaction between the layer and substrate and showed that bonding continues to play a role for the deposition of 2D bonded materials on 3D bonded substrates. This improved understanding of the epitaxy of 2D materials on 3D bonded substrates makes it possible to predict the orientation of 2D materials on conventional substrates. A similar understanding is also desirable for heterostructures grown by vdW epitaxy and the present work is therefore devoted to determine the epitaxial rules for vdW epitaxy.

Instead of studying vdW epitaxy using passivated substrate surfaces, three types of high quality graphene substrates^9^ are used in the present study, Fig. 1(a). The first substrate is the SiC (6√3 × 6√3)R30° buffer layer that consist of a graphene layer that is covalently bonded to the SiC substrate^10^. The second type of substrate is an epitaxial monolayer graphene on top of the SiC (6√3 × 6√3)R30° buffer layer and the third type of substrate is a quasi freestanding bilayer of graphene on top of oxidized SiC^11^. An in depth investigation of the epitaxial monolayer graphene substrate was performed, that revealed that the surface consists of regions covered by the (6√3 × 6√3)R30° buffer layer, monolayer graphene and few layer graphene even though in average only a (6√3 × 6√3)R30° SiC(001) surface reconstruction is observed by reflection high-energy electron diffraction (RHEED). Sb_2_Te_3_ thin films were subsequently grown by molecular beam epitaxy (MBE) and characterized by Raman spectroscopy, which showed that the Sb_2_Te_3_/graphene interface is stable. Finally, atomic force microscopy (AFM) and x-ray diffraction (XRD) studies on the epilayers reveal two distinct nucleation mechanisms of Sb_2_Te_3_ on the graphene. By comparing the in-plane orientation of Sb_2_Te_3_ domains grown on graphene with epilayers grown on the buffer layer and on quasi freestanding bilayer graphene the origins of these different nucleation mechanisms are elucidated. Finally, the implications for the synthesis of vdW heterostructures are discussed.

## Results

**Figure 1:**
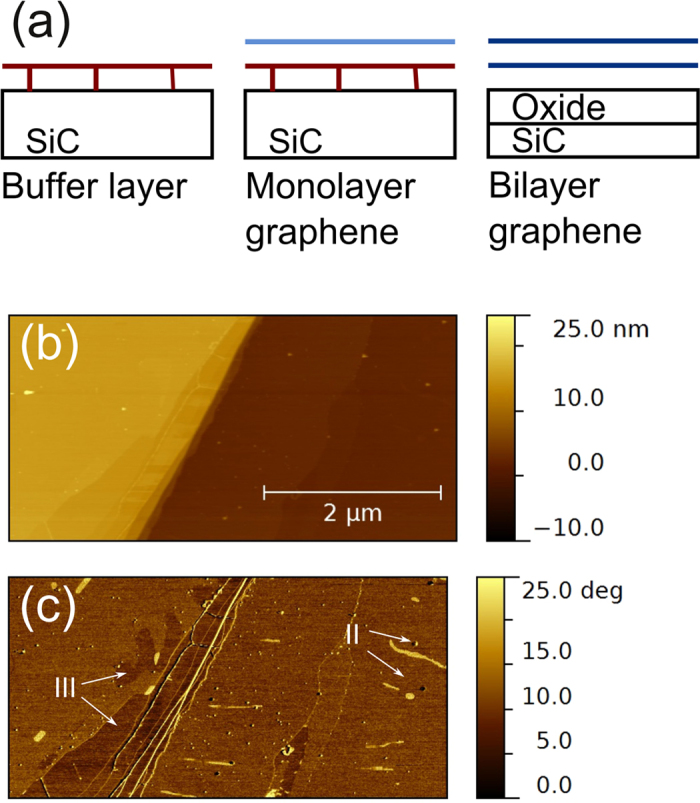
Substrate characterization by AFM. (**a**) Schematic of the substrates used in this study. The red lines represent the buffer layer that is bonded to the substrate, the light blue line represents the monolayer of graphene and the two dark lines represent the freestanding bilayer of graphene. (**b**) AFM topography and (**c**) phase images of a same area of a monolayer graphene substrate. The regions covered by the buffer layer and by few layer graphene are marked by II and III respectively.

Before depositing material on a substrate, it is important to know its topography. Therefore the monolayer epitaxial graphene substrates were studied by tapping-mode AFM. Fig. 1(b), shows that the graphene surface consists of large terraces and that the surface steps, due to the substrate miscut, are bunched together. It was found that the terrace widths typically exceed 2 μm and the step-height ranges from 10 to 15 nm. The phase difference in tapping mode AFM is sensitive to the strength of the inelastic interaction between tip and surface and can thus be used to obtain additional information about the surface chemistry^12^. Figure 1(c) shows the phase image corresponding to the topography image of Fig. 1(b). The phase image exhibits contrast, indicating that the surface consist of areas with a different chemical nature. More specifically, three areas with different amounts of phase differences are observed. The main part of the surface has a phase difference of approximately 11°. Since most of the substrate is covered by graphene, we attribute this phase difference to a graphene surface coverage. In addition, there are zones with a larger phase contrast, indicated by II in Fig. 1(c). These areas are attributed to the presence of residual SiC buffer-layer on the surface, because the SiC buffer-layer displays a larger phase contrast compared to graphene^13^. This is consistent with the observation that these regions are approximately 0.4 nm higher than the surrounding graphene. Finally, the areas close to the step-edge, labeled III, exhibit a smaller phase contrast. It is known that these regions can contain few graphene layers^14^ and that the attractive force of graphene decreases with increasing layer thickness^15^. The areas with the lowest (III) phase contrast are thus those where multilayer graphene prevails. The SiC buffer layer accounts for no more than 5% of the substrate surface, indicating that the substrate has a predominant 2D character.

**Figure 2:**
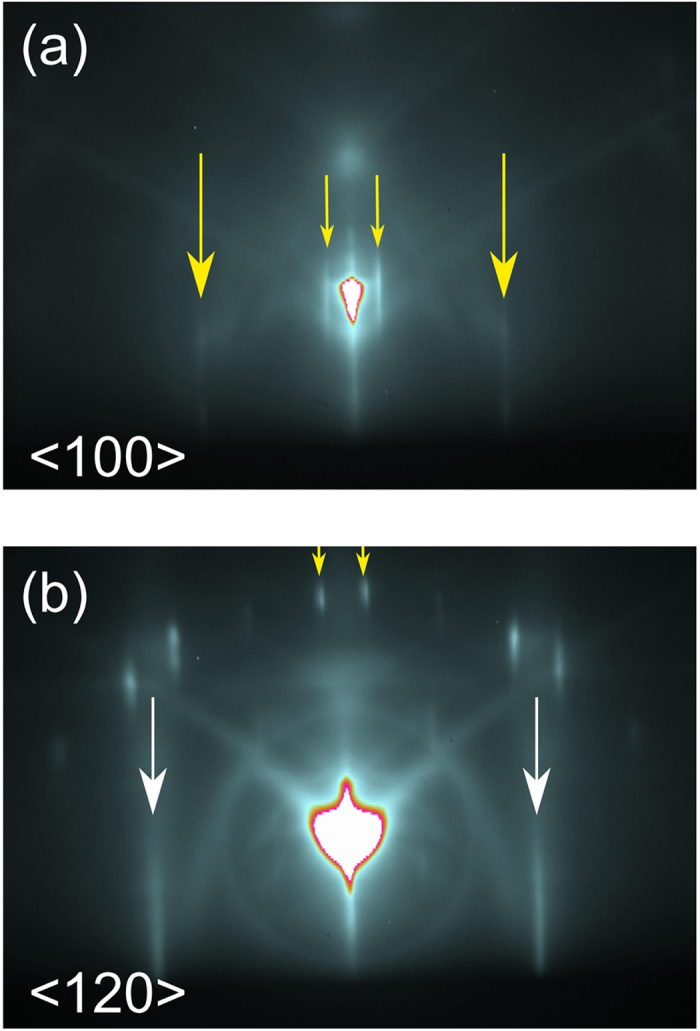
Substrate characterization by RHEED. RHEED images of a monolayer graphene sample taken perpendicular to the SiC < 100 > and < 120 > directions, (**a**,**b**) respectively. The large yellow and white arrows point toward the reflections due to the SiC and graphene in-plane lattice constant, respectively. The small arrows mark the reflections due to the surface reconstruction.

The structural properties of the monolayer epitaxial graphene substrates were studied using RHEED. Diffraction pattern taken perpendicular to the SiC < 100 > and < 120 > directions are shown in Fig. 2(a,b). Both diffraction patterns exhibit strong diffraction streaks, indicated by the large arrows. The spacings between the diffraction peaks in Fig. 2(a) and Fig. 2(b) are however different and the ratio between the spacings is determined to be 1.26 ±0.02. This agrees well with the ratio between the graphene/SiC lattice spacings. Therefore, the diffraction streaks indicated by the white arrows in Fig. 2(b) are ascribed to graphene and the streaks in Fig. 2(a) to the SiC lattice. The observation that the diffraction peaks corresponding to graphene and SiC are observed along different in-plane directions shows that the SiC and graphene lattices are rotated by 30° with respect to each other. A more detailed view of the reciprocal lattice as probed by RHEED can be obtained by studying the higher order diffraction peaks on the different Laue diffraction circles, as indicated by the short yellow arrows. The reconstructed reciprocal lattice, see supplementary information, is found to be consistent with the (6√3 × 6√3)R30° surface reconstruction of SiC^10^. After the structural characterization by RHEED, Sb_2_Te_3_ films were deposited on the graphene surfaces by MBE as described in the methods section.

**Figure 3:**
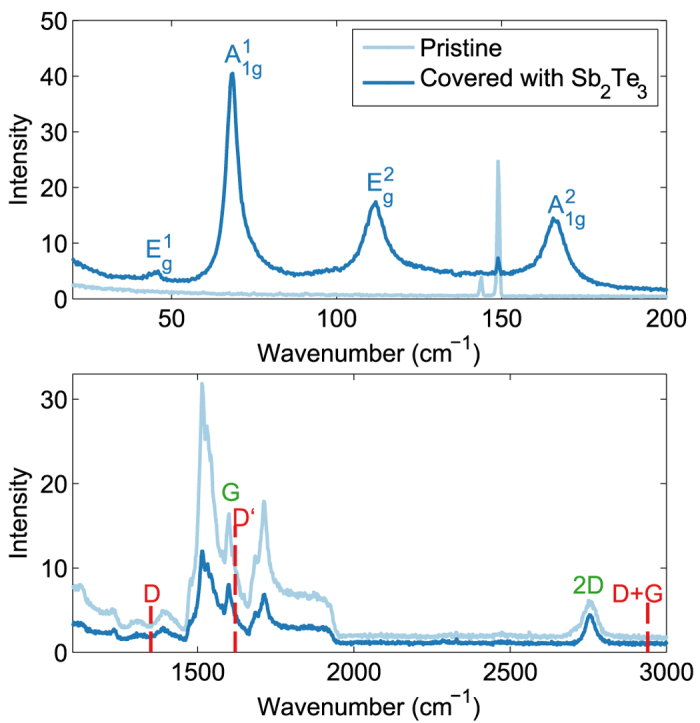
Raman characterization of Sb_2_Te_3_ and graphene covered with Sb_2_Te_3_. Raman spectra of a 11nm thick Sb_2_Te_3_ film grown on monolayer graphene in the spectral ranges relevant for (**a**) Sb_2_Te_3_ and (**b**) graphene taken in regions with and without Sb_2_Te_3_ epilayer. The observed Raman modes of Sb_2_Te_3_ are labeled accordingly in (**a**). The position of the graphene Raman modes (G and 2D) and the graphene Raman modes that are related to defects (D, D’ and D  +  G) are indicated in (**b**).

The stability of an interface is an important aspect of any epitaxial heterostructure. In order to address this topic, Sb_2_Te_3_ layers and monolayer epitaxial graphene substrates were studied using Raman spectroscopy. Figure 3 shows Raman spectra taken on the same sample in a region covered with Sb_2_Te_3_ and a region that intentionally was not covered with Sb_2_Te_3_ during the deposition. The Raman spectra in the range from 20–200 cm^−1^ is shown in Fig. 3(a). For the region that is covered with Sb_2_Te_3_ one can clearly see the presence of the characteristic Raman modes of Sb_2_Te_3_, whereas they are absent in the region not covered by Sb_2_Te_3_. Note that the sharp lines around 150 cm^−1^ correspond to the E_2_ doublet of SiC^16^. Figure 3(b) shows the Raman spectra taken in the spectral range that is relevant for SiC and graphene. The intensity of the Raman signal for the area covered by Sb_2_Te_3_ is clearly lower, which is due to the absorption of the light in the Sb_2_Te_3_ layer. Nevertheless the spectra are almost identical and the G and 2D peaks, characteristic of graphene, can clearly be observed in both spectra. Furthermore, the defect related peaks, i.e. D, D′ and D + G, are not observed in both spectra. We note that these peaks were also not observed after subtraction of the SiC background signal. This indicates that the graphene structure is unaffected by the deposition of the Sb_2_Te_3_ layer and shows that the Sb_2_Te_3_/graphene interface is stable. Moreover, the absence of the defect related peaks is a good indication that there is no rehybridization in graphene and thus that there are no covalent bonds formed between graphene and Sb_2_Te_3_.

**Figure 4:**
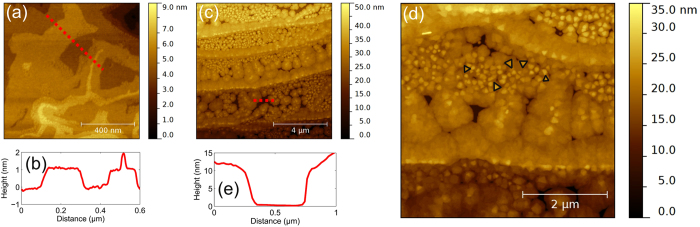
Surface morphology of Sb_2_Te_3_ on epitaxial graphene. (**a**) AFM topography image of 24 nm thick Sb_2_Te_3_ film grown on a monolayer graphene substrate. (**b**) The line profile taken along the dotted line in (**a**) shows that the surface consist of 1 nm steps corresponding to a quintuple layer of Sb_2_Te_3_. (**c**,**d**) AFM topography images taken with different magnifications of an 11 nm thick Sb_2_Te_3_ film grown on a monolayer graphene substrate, displaying different nucleation densities. The small triangles mark some of the different nucleation site with different orientations. (**e**) Line profile taken along the dotted line in (**c**) showing the absence of Sb_2_Te_3_ in some areas of the surface.

Now we turn to the investigation of the surface topography of the Sb_2_Te_3_ films. Figure 4(a) shows a topography image of a Sb_2_Te_3_ film with a thickness of 24 nm. It shows that the surface of the 24 nm thick film consists of large flat areas. Those are characterized by terraces separated by 1 nm high steps, as evidenced by the line profile, Fig 4(b). It should be pointed out that the step-height corresponds to the height of a single quintuple layer, i.e. the basic building block of Sb_2_Te_3_. A more detailed view of the initial growth is obtained by studying surface morphology of an 11 nm thick film, i.e. Fig. 4(c,d). The AFM image in Fig. 4(c) shows that the step-and-terrace structure of the substrate is still clearly visible. Furthermore, the surface exhibits depressions with a depth of approximately 10 nm, Fig. 4(e), which corresponds to the thickness of the film. In addition, the depressions display a phase contrast (image not shown) with the remainder of the film. Therefore, we conclude that there is no Sb_2_Te_3_ present in these areas. Furthermore, in Fig. 4(c) and in the higher magnification image, Fig. 4(d), two different nucleation densities can be observed. The areas with high nucleation density occur on the substrate terraces and seem to be disordered, as is deduced from the position and orientation of the triangular shaped nucleation sites marked in Fig. 4(d). However, the areas with a low nucleation density do show some order, i.e. they align perpendicular to the step-edge and can be found at the step-edges. However, we note that this alignment doesn’t occur on all terraces. The observation of residual buffer layer on the graphene surface, Fig 1(c), that also is aligned perpendicular to the step-edges, suggests that the buffer layer influences the nucleation and growth of the Sb_2_Te_3_ layer. It should be pointed out that this particular alignment of the residual part of SiC buffer layer was not observed on all terraces. These observations are therefore consistent with a preferred nucleation of Sb_2_Te_3_ on the SiC buffer layer as a cause of the observed ordering.

**Figure 5:**
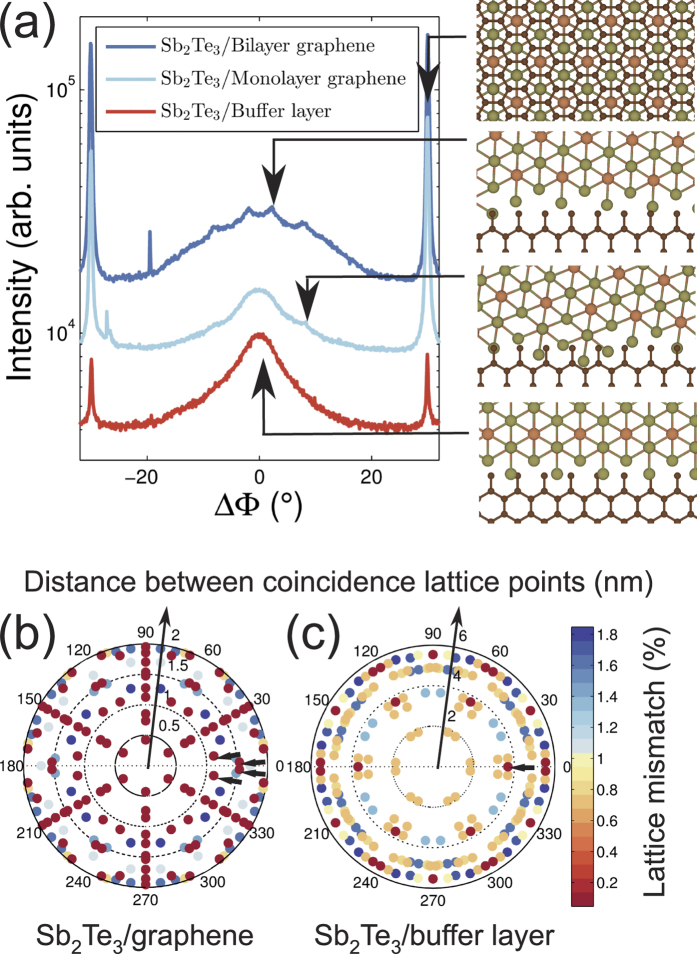
In-plane orientation of Sb_2_Te_3_ on different graphene substrates. (**a**) XRD φ-scans on the Sb_2_Te_3_ (015) diffraction peak for films grown on three different kind of graphene substrates as shown in Fig. 1(a): quasi freestanding bilayer graphene, monolayer graphene and on the SiC buffer layer. The films have thicknesses of 28 nm, 24 nm and 19 nm, respectively. The relative orientation between the Sb_2_Te_3_ film and the graphene layer are indicated in the schematics on the right. C, Te and Sb atoms are represented by brown, green and orange circles, respectively. For clarity reasons the crystal lattices in the lower three top view schematics are not overlapping. The absolute lattice mismatch of Sb_2_Te_3_ as a function of angle and distance between the coincidence lattice sites with respect to (**b**) graphene and (**c**) the corrugations of the buffer layer. For clarity reasons lattice mismatch exceeding 2% are omitted from the graph.

RHEED patterns taken after the growth (see suppl. Fig. S2) consisted of streaks separated by different spacing, indicating that the film consists of domains with different orientations. In order to determine the orientation of these domains, the structural properties were studied in detail using XRD. All the diffraction peaks observed in the linear Q_z_ scan could be indexed as (00n) peaks, indicating that all domains have a (001) out-of-plane orientation. This implies that the domains have a different in-plane orientation. XRD φ-scans on Sb_2_Te_3_ (015) and SiC(013) diffraction peak were performed in order to determine the in-plane orientation. The relative orientation of the Sb_2_Te_3_ layer with respect to the graphene lattice was determined by considering the 30° rotation of the graphene lattice with respect to the SiC lattices as determined by RHEED, Fig. 2. The result is shown in Fig. 5(a) (middle light blue line) and confirms the presence of domains with different in-plane orientation. More precisely, it shows that the Sb_2_Te_3_ layer grown on graphene consists of domains that are rotated by ±30° with respect to the graphene lattice and of domains that are approximately aligned with the graphene lattice. We note that extended φ-scans showed a 6-fold rotational symmetry, indicating that the film is twinned, as expected from symmetry considerations^17^. These observations are in agreement with grazing incidence XRD studies performed on Bi_2_Se_3_ layers grown on graphene^18^,^19^. However, the φ-scans presented here also reveal some additional details that were not previously observed: a close inspection of the broad feature in the φ-scans reveals that it consists of multiple peaks. The additional peaks, indicated by the arrows, have a separation of ±2° and ±8° from 0°, indicating that some domains have a well defined twist angle with respect to the graphene lattice. The different epitaxial relationships between the graphene and Sb_2_Te_3_ that are observed are illustrated in the schematics in Fig. 5(a).

The observation of rotational twists is often related to the formation of a coincidence lattice between substrate and epilayer. In order to indentify if this is the case, the lattice mismatch between graphene (a = 2.46 Å) and Sb_2_Te_3_ (a = 4.26 Å) was calculated as a function of the rotational angle and the distance between the coincidence lattice points by adapting the approach of Kaneko *et al*^20^. to hexagonal lattices. The resulting polar plot in Fig. 5(b) shows that multiple orientations result in lattice mismatches as low as 0.02%. The highest density of coincidence lattice points for this lattice mismatch is obtained when the Sb_2_Te_3_ lattice is rotated with 30° with respect to graphene. Thus one can expect that this orientation is the most pronounced one, which is consistent with the experimental data. Furthermore, it is observed that a coincidence lattice with the same lattice mismatch can be formed when the rotation between the layers is 2.2° or 8.2° (indicated by the small arrows in Fig. 5(b)), although the distance between coincidence lattice sites is larger. These two angles perfectly match with the experimentally observed angles, confirming that coincidence lattices are formed between graphene and Sb_2_Te_3_. The epitaxial relationship between Sb_2_Te_3_ and monlayer epitaxial graphene can thus be understood to a large degree by considering their in-plane lattice constants.

Besides the orientations that are due to the formation of coincidence lattices, a large part of the Sb_2_Te_3_ layers grown on monolayer epitaxial graphene has an orientation that is approximately aligned with the graphene lattice. This suggests that there is another mechanism that influences the in-plane orientation of Sb_2_Te_3_. This is consistent with the AFM observations showing that there are two different nucleation densities, and that the growth of Sb_2_Te_3_ is affected by the SiC surface regions covered by the (6√3 × 6√3)R30° buffer layer. It is thus important to address the influence of these regions on the epitaxial relationship between Sb_2_Te_3_ and graphene. Therefore, additional Sb_2_Te_3_ films were grown on SiC substrates mainly covered with the SiC (6√3 × 6√3)R30° buffer layer and on quasi freestanding bilayer graphene substrates. The XRD φ-scan performed on the film grown on the buffer layer substrate, Fig. 5(a) (lower red line), shows that the sharp features at ±30° are reduced in intensity, whereas the intensity of the broad feature around 0° is increased. Furthermore, the additional peaks ±2° and ±8° are no longer observed. On the other hand, the peaks at 2° and 8° become clearer for the growth on the quasi freestanding bilayer graphene, Fig 5(a) (upper dark blue line). Based on these observations, it is now possible to attribute the growth on the SiC (6√3 × 6√3)R30° buffer layer to the broad feature at 0° and the formation of coincidence lattice to the growth of Sb_2_Te_3_ on mono-/bilayer graphene.

However, it is unclear why the growth on the buffer layer differs so much from the two other cases given that they are topologically identical^9^,^21^. Recent experiments showed that Sb_2_Te_3_ although a 2D material still can form a coincidence lattice with a reconstructed surface^8^. We therefore considered the possibility of the formation of a coincidence lattice between the SiC 6√3 × 6√3R30° surface reconstruction and Sb_2_Te_3_. The chemical bonding between SiC and the buffer layer results in a (6 × 6) corrugation of the buffer layer. Such a corrugation changes the distance between the carbon and Sb_2_Te_3_ layers periodically and hence might influence the interfacial interaction. Therefore, the lattice match of coincidence lattices between Sb_2_Te_3_ and the (6 × 6) corrugation were calculated, Fig. 5(c). As expected the distance between the coincidence lattice points increases compared to the graphene case due to the larger surface unit of the buffer layer. Furthermore, it is observed that the minimum distance between the coincidence lattice points having a lattice mismatch <0.1% is obtained when the Sb_2_Te_3_ and the carbon lattice are not rotated with respect for each other. Based on these calculation one thus would expect an alignment of the carbon and Sb_2_Te_3_ lattices, which is in full agreement with the experimental results. It is therefore concluded that the epitaxial alignment between Sb_2_Te_3_ and the buffer layer is due to the formation of a coincidence lattice between the corrugations of the buffer layer and Sb_2_Te_3_.

Based on these considerations, we conclude that the different nucleation mechanisms of Sb_2_Te_3_ on graphene substrates, Fig. 4, and the observation of Sb_2_Te_3_ domains that are aligned with the graphene lattice, Fig 5(a), are due to an imperfect surface coverage of the monolayer graphene sample, as indeed observed by AFM, see Fig. 1(b,c). We note however that the surface area that is covered by the buffer layer accounts for no more than 5% of the surface area, whereas the amount of Sb_2_Te_3_ domains that are aligned with the buffer layer account more than 70% of the total amount of domains. This indicates that the amount of domains with a certain orientation is not only affected by the surface chemistry, but also by the growth mode and growth rate of the nucleated layers.

## Discussion

The observation of coincidence lattices between two 2D bonded materials indicates that there is a significant interaction between Sb_2_Te_3_ and the substrates. If this interaction is solely due to vdW bonding or also involves chemisorption, as for example observed at a hetero-organic interface^22^, remains an open question. Furthermore, it is unclear why the coincidence lattices rotated by 2.2° or 8.2° are formed at all when Sb_2_Te_3_ is deposited on monolayer epitaxial graphene and quasi freestanding bilayer graphene, because the same lattice mismatch can be obtained with a much higher density of coincidence lattice sites (rotated by 30°). A possible explanation for this observation is that the relatively weak interaction between the layers reduces the influence of the density of coincidence lattice points on the energy difference between the different orientations. This makes the occurrence of coincidence lattice with large distances between coincidence lattice sites more likely. However, the exact nature and the energetics of bonding between 2D bonded materials is still unclear, as pointed out above, and requires more detailed investigations. Nevertheless, these observations do show that lattice matching still plays an important role during vdW epitaxy and even suggest that coincidence lattice formation might be natural for vdW epitaxy on 2D bonded materials. This implies that the lattice parameter of 2D bonded materials is an important parameter to consider for the design and fabrication of vdW heterostructures.

In summary, the fundamentals of vdW epitaxy were studied by using Sb_2_Te_3_ films grown on three different kind of graphene substrates: monolayer epitaxial graphene substrates, quasi freestanding bilayer graphene substrates and on the SiC buffer layer substrates. Raman spectroscopy revealed that graphene forms a stable interface with Sb_2_Te_3_. Furthermore, it was shown that the in-plane alignment between substrate and epilayer is determined by the formation of coincidence lattices between Sb_2_Te_3_ and graphene (monolayer and bilayer graphene substrates) or the surface corrugations of the buffer layer, demonstrating that there is a significant interaction between 2D materials and that lattice matching still occurs during vdW epitaxy. Contrary to early reports on vdW epitaxy^7^ that suggested a reduction of the lattice matching condition, these results thus clearly underline the fact that lattice matching still plays an important role during vdW epitaxy. These results thus provide necessary guidelines for the improvement and realization of complex vdW heterostructuress by vdW epitaxy.

## Methods

Sb_2_Te_3_ thin films were deposited on three different kind of graphene substrates (monolayer graphene, bilayer graphene and the SiC buffer layer) using molecular beam epitaxy (MBE). The fabrication and characterization of epitaxial graphene, bilayer graphene and buffer layers on top of SiC(001) substrates used in this study is described in detail elsewhere^9^. Before introducing the substrates in the growth chamber they were outgassed at 350 °C in the preparation chamber. In order to determine a suitable deposition temperature the binding energy between Sb_2_Te_3_ and graphene was considered. Using density functional theory Jin and Jhi calculated the binding energy between graphene and Sb_2_Te_3_ and found it to be ~41 meV^23^. This is equal to the thermal energy, k_B_T, where k_B_ is Boltzmann’s constant and T the temperature, when T = 475K/ 202 °C. The binding energy thus puts an upper limit on the deposition temperature that can be used. Indeed, we observe a total desorption from the graphene surface for temperatures above 150 °C. Initial deposition with a growth temperature of 135 °C, which is approximately 100 °C below normal deposition conditions^8^, contained a large amount of Te participates. In order to overcome this problem a deposition scheme was adopted similar to that used for Bi_2_Se_3_ films^24^.

The substrates and films were characterized *in situ* using RHEED using a 20 keV electron acceleration voltage and *ex situ* using AFM (Veeco Nanoscope III) operated in tapping mode. and XRD (PANalytical X’Pert Pro) using Cu Kα_1_ radiation (λ = 1.540598 Å). The Raman spectra were obtained by exciting the Sb_2_Te_3_ and graphene/SiC with a laser (λ = 633 nm and λ = 473 nm, respectively). The backscattered light was collected using a liquid nitrogen cooled charged coupled device detector without using any polarizer’s. The schematics in the Fig. 5(a) are produced using VESTA^25^.

## Additional Information

**How to cite this article**: Boschker, J. E. *et al.* Coincident-site lattice matching during van der Waals epitaxy. *Sci. Rep.* **5**, 18079; doi: 10.1038/srep18079 (2015).

## Supplementary Material

Supplementary Information
