# Supplementary material for: Coincident-site lattice matching during van der Waals epitaxy
Source: Sci Rep. 2015 Dec 14;5:18079. doi: 10.1038/srep18079 (PMC4677287; doi:10.1038/srep18079)
Supplement: Supplementary Information [file srep18079-s1.pdf]

Supplementary information for

Coincident-site lattice matching during van der Waals epitaxy

Jos E. Boschker, Lauren A. Galves, Timur Flissikowski, J. Marcelo J. Lopes, Henning Riechert, and Raffaella Calarco

Paul-Drude-Institut für Festkörperelektronik, Hausvogteiplatz 5-7, 10117 Berlin, Germany, EU

RHEED characterization

Graphene/SiC surface reconstruction

The RHEED patterns of the graphene substrate shown in the main text show a number of higher order diffraction peaks. Starting with the higher order diffraction peaks (short yellow arrows) in Fig 2(a) in the main text it is calculated that they correspond to 1/6 of the SiC reciprocal lattice spacing (long yellow arrows). From the positions of the higher order diffraction peaks it is possible to reconstruct the reciprocal lattice of the surface. To this end, the diffraction peaks observed along the <100> direction are indicated as blue dots in suppl. Fig. S1. RHEED patterns acquired with higher intensity (not shown) revealed that the 1st and 2nd order Laue circles are particularly low in intensity, whereas some peaks on the 3rd order Laue circle could be discerned. The 3rd order Laue circle corresponds to a cut through reciprocal space at an offset in the <120> direction, as indicated in suppl. Fig. S1 together with the observed diffraction peaks on this Laue circle. In a similar fashion the higher order peaks observed on the 0th, 1st and 2nd order Laue circles observed with the electron beam perpendicular to <120>, i.e. Fig. 2(b) of the main text, can be plotted in suppl. Fig. S1. By taking the symmetry of the surface into account the total reciprocal lattice can be reconstructed. This approach makes it also possible to visualize the reciprocal lattice around the origin that normally is not observable by other techniques such as low energy electron

diffraction. The reconstructed reciprocal lattice, see suppl. Fig. S1, is consistent with the (6√3×6√3)R30° surface reconstruction of SiC.

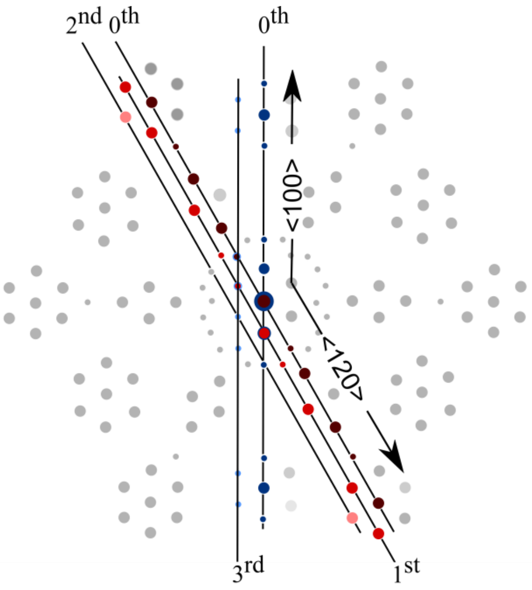

Supplementary Figure S1: Schematic of the reconstructed reciprocal lattice of epitaxial graphene. Red and blue dots represent diffraction peaks observed along the <120> and <100> in-plane directions. The positions of the grey dots are obtained from symmetry considerations.

Sb_2Te_3 film on graphene

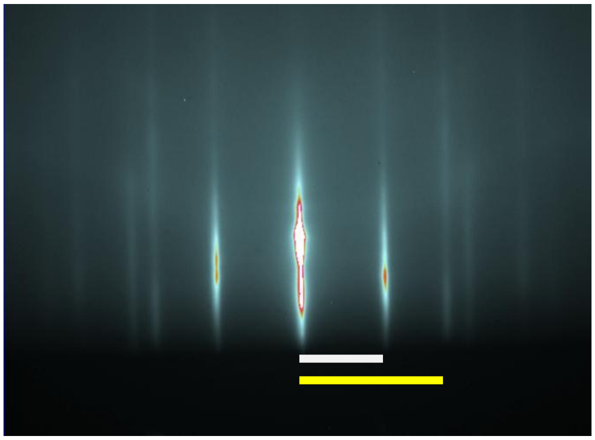

Supplementary Figure S2: RHEED pattern after growth. RHEED pattern of a 24 nm thick Sb_2Te_3 film grown on epitaxial graphene substrate. The yellow and white bars mark RHEED streaks separated by different spacing's, indicative of domains with different in-plane orientations.
